# Strengths and limitations of relative wealth indices derived from big data in Indonesia

**DOI:** 10.3389/fdata.2023.1054156

**Published:** 2023-02-21

**Authors:** Daniele Sartirano, Kyriaki Kalimeri, Ciro Cattuto, Enrique Delamónica, Manuel Garcia-Herranz, Anthony Mockler, Daniela Paolotti, Rossano Schifanella

**Affiliations:** ^1^ISI Foundation, Turin, Italy; ^2^UNICEF, New York, NY, United States; ^3^UNICEF, Jakarta, Indonesia; ^4^Department of Computer Science, University of Turin, Turin, Italy

**Keywords:** wealth, index, poverty, survey, machine learning

## Abstract

Accurate relative wealth estimates in Low and Middle-Income Countries (LMICS) are crucial to help policymakers address socio-demographic inequalities under the guidance of the Sustainable Development Goals set by the United Nations. Survey-based approaches have traditionally been employed to collect highly granular data about income, consumption, or household material goods to create index-based poverty estimates. However, these methods are only capture persons in households (i.e., in the household sample framework) and they do not include migrant populations or unhoused citizens. Novel approaches combining frontier data, computer vision, and machine learning have been proposed to complement these existing approaches. However, the strengths and limitations of these big-data-derived indices have yet to be sufficiently studied. In this paper, we focus on the case of Indonesia and examine one frontier-data derived Relative Wealth Index (RWI), created by the Facebook Data for Good initiative, that utilizes connectivity data from the Facebook Platform and satellite imagery data to produce a high-resolution estimate of relative wealth for 135 countries. We examine it concerning asset-based relative wealth indices estimated from existing high-quality national-level traditional survey instruments, the USAID-developed Demographic Health Survey (DHS), and the Indonesian National Socio-economic survey (SUSENAS). In this work, we aim to understand how the frontier-data derived index can be used to inform anti-poverty programs in Indonesia and the Asia Pacific region. First, we unveil key features that affect the comparison between the traditional and non-traditional sources, such as the publishing time and authority and the granularity of the spatial aggregation of the data. Second, to provide operational input, we hypothesize how a re-distribution of resources based on the RWI map would impact a current social program, the Social Protection Card (KPS) of Indonesia and assess impact. In this hypothetical scenario, we estimate the percentage of Indonesians eligible for the program, which would have been incorrectly excluded from a social protection payment had the RWI been used in place of the survey-based wealth index. The exclusion error in that case would be 32.82%. Within the context of the KPS program targeting, we noted significant differences between the RWI map's predictions and the SUSENAS ground truth index estimates.

## 1. Introduction

The UN Sustainable Development Goals calls for ending poverty in all forms by 2030. Household monetary poverty has devastating effects, particularly on children, concerning health (Brooks-Gunn and Duncan, 1997) and education (Lacour and Tissington, 2011). National governments seek to increase universal social welfare with targeted money transfers (Daimon, 2001), particularly in low and medium-income countries such as Togo (Aiken et al., 2022), Indonesia (Alatas et al., 2016), or Zambia (Brady, 2013). Social protection programs should be universal, capable of implementing nationally appropriate social protection systems and measures for all and, by 2030, achieve substantial coverage of the poor. Yet, sometimes, targeting mechanisms are short-term and temporary solutions due to budget and resource constraints. The selective targeting approach introduces potential inclusion and exclusion errors, with inevitable trade-offs among equity, effectiveness, and efficiency (Cornia and Stewart, 1993; Hanna and Olken, 2018).

Targeting policies are traditionally based on estimating proxies to identify households and individuals satisfying low-income levels. Surveys provide nationally representative estimates of poverty and income distribution. However, their strength (i.e., accuracy and reliability) is based on randomly capturing a population sample and asking many questions. When targeting based on income characteristics, information about all of the population—or at least the potential candidates to receive a cash transfer or other benefits—is needed. Thus, it is not possible to replicate the accuracy of the survey and proxies are used to try and assess the income level of households or individuals using short questionnaires. As these proxies often incur errors, it has often been argued that a categorical approach (e.g., based on age or geographic location) is less costly, fairer, and more accurate. In recent years, scholars and policymakers are increasingly becoming interested in alternative digitally-available data sources, such as social media data or satellite images, that, coupled with machine learning-based approaches, make it possible to infer advanced socio-demographic attributes (Jean et al., 2016; Engstrom et al., 2017; Adler et al., 2019; Kalimeri et al., 2019; Rama et al., 2020; Yeh et al., 2020). Such approaches allow for fast, fine-grained spatial resolutions, providing something similar to a real-time digital population census. Given the social implications, such methods should be thoroughly evaluated before informing decision-making, impacting social welfare.

In this work, we focus on Indonesia, a country with an estimated population of 270 million, as an example to assess the advantages and limitations of using this alternative type of data. Indonesia's poverty rate based on the national monetary poverty line reached a record low of 9.2% in September 2019.^1^ The share of Indonesians living below the national poverty line has more than halved since 1993.^2^ Despite this progress, the pace of poverty reduction post-2010 has been about one-half (0.3% points per year) of what it used to be in 2003–2010 (0.6% points per year). Vulnerability remains high; in 2018, 73.9 million individuals (30% of the population) were either poor or vulnerable to falling back into poverty.^3^ As the Demographic and Health Surveys (DHS) do not have information on income and expenditures, wealth index (Filmer and Pritchett, 2001) is employed. However, although wealth and income are correlated, they are pretty heterogeneous, and the level of correlation is often low. Hence, we first estimate Indonesia's Relative Wealth Index (RWI) by employing two nationwide surveys, namely, DHS and the National Socioeconomic Survey (SUSENAS). Then, we compare them against the RWI proposed in Chi et al. (2022) based on non-traditional digital data, e.g., high-resolution satellite imagery, data from mobile phone networks, topographic maps, and aggregated and de-identified connectivity data from Facebook. A potential geographical targeting based on the RWI map could be an innovative and cost-effective alternative to implement a welfare poverty targeting program. Here, we assess the trade-offs of a potential application of the RWI for policy-making. Nevertheless, it has to be remembered that in Indonesia, targeting is not based on the wealth index but income (Kidd et al., 2020).

Our findings show that the development of a wealth index based on non-traditional sources, although promising, entails pitfalls with significant potential social impact, given the RWI index's high spatial and time sensitivity. Part of the problem is that the wealth index is not stable, i.e., the items selected for estimating the wealth index may introduce noise and significantly alter the results. In this work, we provide an in-depth analysis of the index creation implications and a potential real-life scenario of an actual population targeting program for money transfer in Indonesia.

## 2. Materials and methods

Three umbrella definition categories describe monetary poverty as (1) having less than an objectively defined absolute minimum, (2) having less than others in society, or (3) feeling you do not have enough to get along (Hagenaars and De Vos, 1988). While the latter is subjective, the first two categories can be quantitatively measured *via* a direct metric or a proxy, as described above. The direct measurement of income distribution and monetary poverty is usually done with (1) assets (owned by the household), (2) income (of the household's dwellers), or (3) expenditure (average monthly expenditure of the household). There are household surveys which do not have information on income and expenditure but capture the presence of various (often a significant amount) physical assets in the household. This information can be used to construct a wealth index (which does not capture all wealth as there is no information on savings accounts, property owned in other locations, luxury items like art pieces or gold, and financial assets like stocks or bonds). These are computed at the individual level or, more commonly, at the household level and are often employed as a numerical proxy for the household unit's Socioeconomic Status (SES). In this work, we rely on this type of assets-based definition of wealth and in the following, we describe the data and aggregation used to extract a wealth distribution index from the survey data.

### 2.1. Data sources

#### 2.1.1. Demographic and health surveys

The Demographic and Health Survey (DHS) program has collected representative demographic and health-related data in over 90 countries since the 1980s. While initially, DHS dealt with health-related matters alone, more recently, it integrated wealth index estimates at the highest sub-national administrative level (i.e., the provinces, in the case of Indonesia). For this analysis, we calculate relative wealth using the Filmer-Pritchett (Filmer and Pritchett, 2001) pipeline, allowing direct comparison to the RWI map.

The items considered for the construction of the index describe access to goods such as a source of drinking water, type of toilet, sharing of toilet facilities, the material of the main floor, walls, roof, cooking fuel, household services and possessions, such as electricity, TV, radio, watch, types of vehicles, agricultural land size owned, type and number of animals owned, bank account, types of windows (Rutstein, 2015).

Over the years, the survey's composition slightly changed, question- and answer-wise, and differences in temporal and spatial resolutions are also present across countries and editions.

For example, when monitoring displacement, only the 2002 DHS survey comes with a fine geographical resolution, providing the exact position of each interviewed household within a 2–5 km range, otherwise the spatial resolution is provided at a province level for privacy-preserving issues.

#### 2.1.2. National socioeconomic survey

The National Socioeconomic Survey (SUSENAS) is a household survey carried out by the Indonesian National Statistical Office, Badan Pusat Statistik (BPS), every 1–2 years since 1963–1664, reporting the status of the Indonesian regencies. A regency (or *kabupaten* in Indonesian) is the second administrative division in Indonesia below the province (or *provinci* in Indonesian). In this work, we consider only the latest survey in 2020, which reports 513 of the 522 regencies in Indonesia.

The questions collected refer both to the individual and household level; they cover specific assets within the dwelling, health-related information, education and income level of the family units, as well as the fruition of several Indonesian national programs designed to combat poverty and promote schooling (e.g., cash and food transfers). The spatial resolution is higher than the one obtainable with the DHS data since each household is geographically assigned to a regency. The SUSENAS survey is weighted to represent the country's population at the regency level.

Unlike DHS, in SUSENAS, there is no asset-based wealth index estimation; hence, we engineered an index that serves as a benchmark for the RWI map. This process is described in Section 2.2.

#### 2.1.3. Relative wealth index

The Relative Wealth Index (RWI) (Chi et al., 2022) is an index estimated by a machine learning model for 135 low and middle-income countries to provide micro-estimates (projections) of wealth and poverty at fine-grained 2.4 km resolution tiles. The model was trained on vast and heterogeneous datasets from satellites, mobile phone networks, topographic maps, as well as aggregated and de-identified connectivity data from Facebook. The ground truth measurements of household wealth are collected through traditional face-to-face surveys, following the Filmer-Pritchett (Filmer and Pritchett, 2001) methodology on the DHS 2002 survey. The approach for creating the RWI map overcomes essential limitations of the traditional surveys, such as fine-grained coverage, and timely and cost-efficient data, while extending to countries where DHS does not operate. These estimates are provided free for public use.^4^

### 2.2. Wealth index estimation from SUSENAS

The DHS and RWI wealth index estimates are obtained by applying the Filmer-Pritchett methodology (Filmer and Pritchett, 2001) to a subset of the survey's items and share common strengths and shortcomings. First and foremost, they are *relative* indices meaning they can only be employed to compare the wealth of two areas within the same country. Moreover, their estimates are not absolute numbers and neither comply with a predefined mathematical relationship; for instance, a tile with RWI = 4 is richer than a tile with RWI = 2 but there is no information on how much richer.

We adopt the Filmer-Pritchett methodology to estimate a wealth index at the household level from the SUSENAS survey, mimicking the pipeline employed for DHS (Rutstein, 2015), which is adopted mainly in literature for the same purpose (Vyas and Kumaranayake, 2006). Before that, we implement the following initial steps that are of fundamental importance since even small modifications can alter the results significantly:

- **Item selection:** The choice of assets that are the most relevant for the wealth estimates. This is usually done with input from domain experts. In our case, the item selection was based on previous literature.
- **Data preprocessing:** Handling missing answers and transforming the data into a binary matrix. Households with missing items were excluded from the pipeline; numeric answers were converted to quintiles, except for the number of TVs in the household; “does not know” items were treated separately.

To benchmark, the RWI map as close as possible to its estimates, our ground truth index, which we call *SUSENAS index*, is constructed following a similar methodology to the evaluation of the RWI map (Chi et al., 2022). Similar to Chi et al. (2022), we consider only a subset of 15 items.^5^

### 2.3. Spatial aggregation

Indonesian administrative spatial units consist of 34 provinces which can be further divided into 522 units (between cities and regencies), 513 of which have households interviewed as part of the SUSENAS survey. Differently, the RWI map is provided at a resolution of 2.4 km tiles and aggregated using a population-weighted average. To directly compare the DHS and SUSENAS index wealth index to the RWI map, we need to aggregate to the exact spatial resolution while weighting with the respective population averages.

We employ the High-Resolution Settlement Layer (HRSL) map (Tiecke et al., 2017) to obtain the population estimates per geographic tile. These estimates averaged on the appropriate spatial resolution are applied to weight the indices to the province and regency levels, respectively.^6^

As mentioned above, the DHS surveys, while gathered at the household level, are available at the province level only. At the same time, the SUSENAS indices can be estimated both at province and regency levels. The DHS data are already weighted to render the survey demographically representative; hence, a straightforward averaging at the province level is performed. To estimate the aggregated SUSENAS wealth index, we employed the internal survey weights provided by the BPS. For the SUSENAS index, an administrative region estimate was computed by aggregating the relative wealth indices of the households within that administrative area through a weighted average, where the weights are the ones internally assigned to each household by the SUSENAS survey.

### 2.4. Targeting for cash transfer

#### 2.4.1. Population bias

The Indonesian government has been implemented the Kartu Perlingudan Sosial (KPS) program to combat monetary poverty. According to the official statistics, the KPS program targets households within the 14 poorest percentiles, providing them with money transfer assistance and other economic benefits. In this work, we evaluate the potential socioeconomic impact of adopting the RWI map to guide such program by simulating the targeting of eligible households in the case of the KPS program. Realistically, the targeting for a real-world aid program would consider different data sources and methodologies. The KPS program attempts to identify (and thus benefit) the monetary poor using proxy for income. However, we focus on assessing the match between the RWI and wealth indices based on physical assets in the household. As in Aiken et al. (2022), the RWI map-based geographical targeting is only one step of a more complex pipeline. Still, since our primary goal is to benchmark the map, we assess the impact of its deployment as part of a targeting pipeline compared to a traditional survey estimate. The KPS program specifics are still helpful in providing meaningful budget-related thresholds for this process.

According to Kidd and Diloá (2019), to directly confront the exclusion error of the indices, we aggregate them at the regency level and then divide them into percentiles. Then, we compare the 14%^7^ of the regencies emerging as the poorest (in terms of their relative ranking in ownership of physical assets) from both indices. This approach evaluates the extent to which the RWI map and the ground truth *SUSENAS index* indicate the same regencies to belong to the 14% poorest quintiles (i.e., with the least physical assets).

Secondly, we assess the extent to which the two indices can predict the regencies in which the 14% poorest live. In this case, the indices are iteratively predicting the poorest regency, stopping when adding a regency's population would surpass the 14% threshold of the total Indonesian population. Moreover, we directly evaluate the predictions of both the RWI and SUSENAS index in terms of precision, percentage of exclusion error and percentage of the excluded population, as follows:


(1) $$\begin{array}{l} Precision=\frac{TP}{TP+FP} \end{array}$$

(2) $$\begin{array}{l} Excl_{num}=\frac{FN}{GTR}*100\% \end{array}$$

(3) $$\begin{array}{l} Excl_{pop}=\frac{Pop_{FN}}{Pop_{GTR}}*100\% \end{array}$$

where TP = true positives, TN = true negatives, FP = false positives and FN = false negatives, *Pop*_*FN*_ the total population living in administrative areas labeled as FN, *GTR* is the number of administrative areas to be targeted according to the selected ground truth and *Pop*_*GTR*_ their total population.

#### 2.4.2. Geographic bias

A shortcoming of the previous evaluation is that it considers the regency as the lowest geographical unit for targeting households. This approach entails an intrinsic error. The significant wealth heterogeneity within regencies means that only some households within a particular regency are equally poor. In place of conducting the KPS targeting simulation using a regency by regency method, we directly utilize the household level estimates of the SUSENAS ground truth index. In particular, we compute how many people each interviewed household represents by considering the household weights as follows:


(4) $$Pop_{hh}=\frac{FWT_{hh}}{\sum_{hh\subset reg}FWT_{hh}}Pop_{reg}$$

where *hh* is the interviewed household, *reg* the regency, *Pop* stands for the population and *FWT* is the household-level weight assigned by the SUSENAS survey.

## 3. Results and discussion

To address our primary research question, i.e., whether we can trust a machine learning based wealth index for social assistance, we faced several theoretical and technical issues. Before delving into evaluating the RWI map, we highlight blind spots and shortcomings of the traditional survey data available to policy makers.

### 3.1. Wealth index estimation

**Figure 1:**
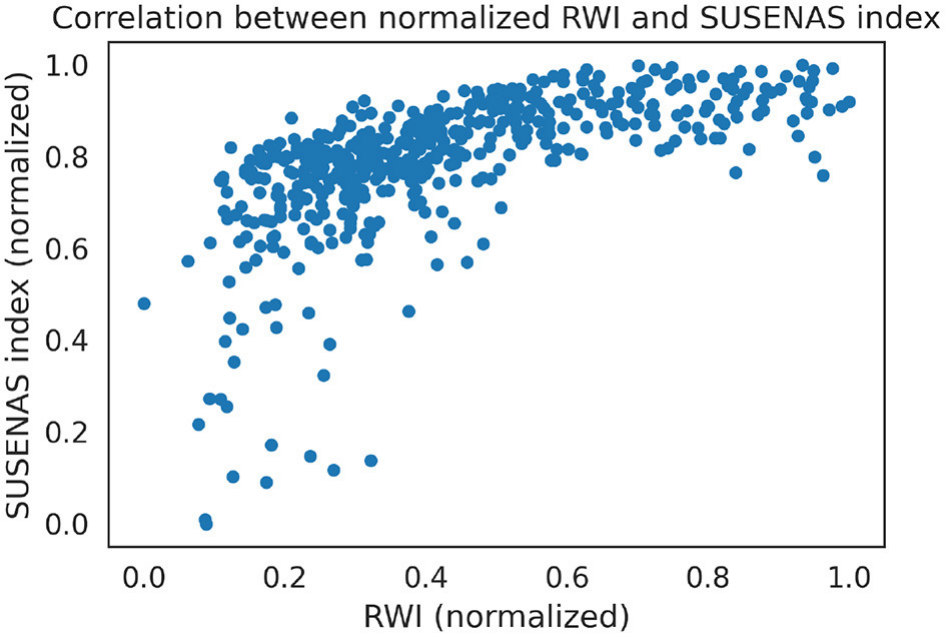
We plot the regency-level estimates for both RWI and the SUSENAS index. The indices are both normalized to be between 0 and 1. We can see that, except for outliers among the poorest regencies, the two estimates are significantly correlated.

Through the methods described in Section 2.2, we estimated the wealth indices for DHS and SUSENAS and compare with the RWI map estimates by means of Spearman correlation. The RWI map was initially trained on the 2002 DHS estimates and correlate well also with the SUSENAS index (ρ = 0.75, *p* < 0.001), as shown in Figure 1. The Spearman index shows good performances even in those regions that were not present in the 2002 DHS: computing the Spearman ranking correlation coefficient in those regions only, we find (ρ = 0.70, *p* = 10^−13^). This means that the RWI map maintains a good performance in ranking the regencies situated in those regions where no households were interviewed as part of the DHS 2002 survey.

### 3.2. Spatial aggregation

Geographical targeting is a commonly employed approach in social assistive programs, however its performance is strongly affected by the spatial variability, which we now consider.

**Figure 2:**
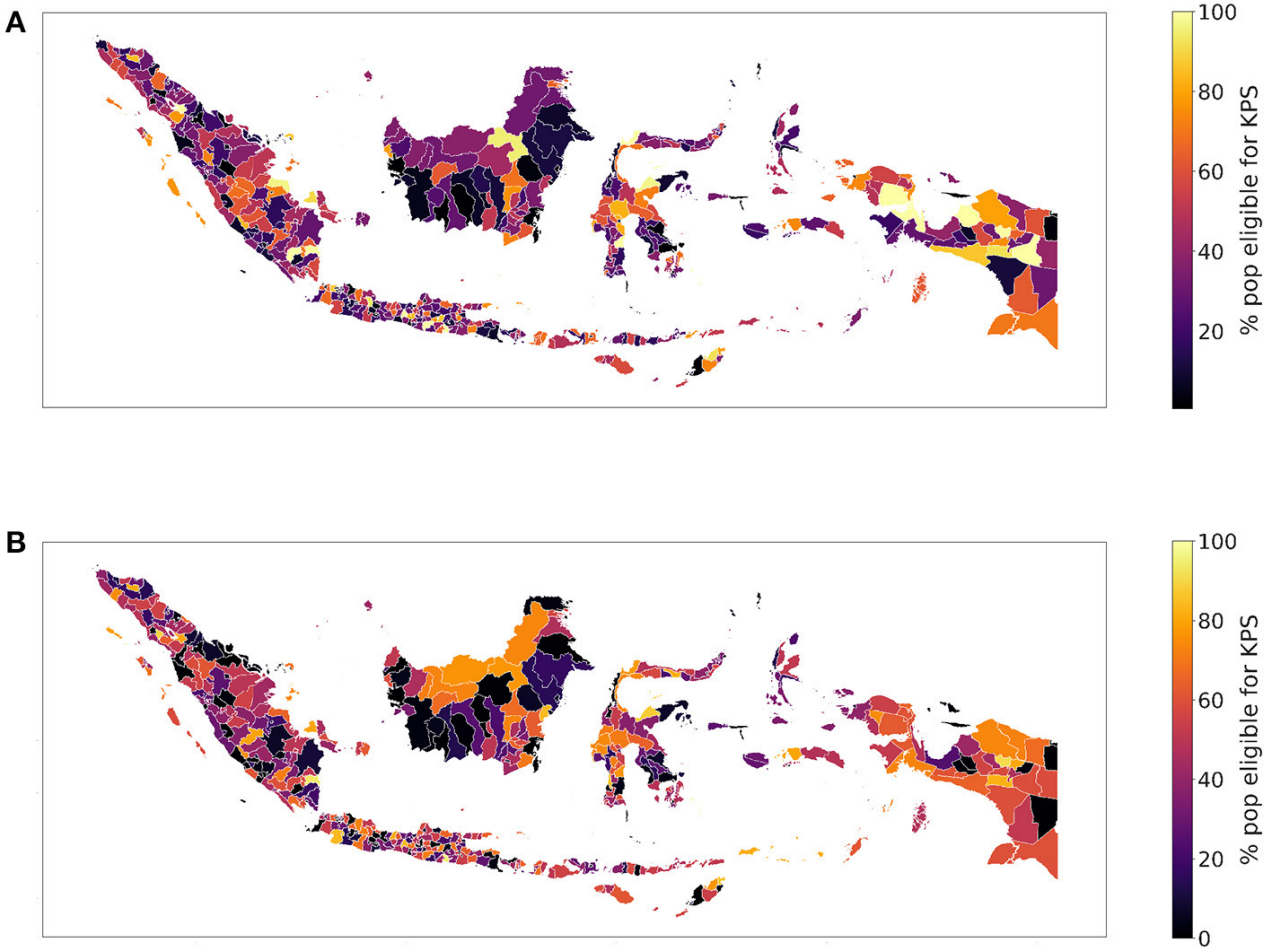
We depict the percentage of people belonging to the poorest quintile according to the **(A)** SUSENAS-engineered wealth index or **(B)** RWI map estimates, aggregated at the regency level. A higher percentage corresponds to a poorer regency.

According to Fry et al. (2014), we compute the percentage of the poorest individuals within a regency. Visualizing the two indices, we obtain slightly different maps; according to the SUSENAS index Nduga (in Papua) is the poorest regency while Tangerang Selatan (in Jakarta, Java) is the richest (particularly Jakarta and the nearby regencies), as shown in Figure 2A. Instead, according to the RWI in Figure 2B, Mamberamo Raja (in Papua) is the poorest regency while Center Jakarta (in Java) is the richest. We also notice that the SUSENAS index predicts a significant amount of regencies to be close to fully inhabited by people living in the poorest quintile, while this does not happen with the RWI map. This is probably due to the higher geographical precision of the latter, which manages to better show the spatial heterogeneity of the wealth distribution. However, this added precision may also lead to unintended drawbacks, such as psycho-social costs of targeting (Devereux et al., 2015). Since the indices are relative by definition, the ranking may alter the results.

**Figure 3:**
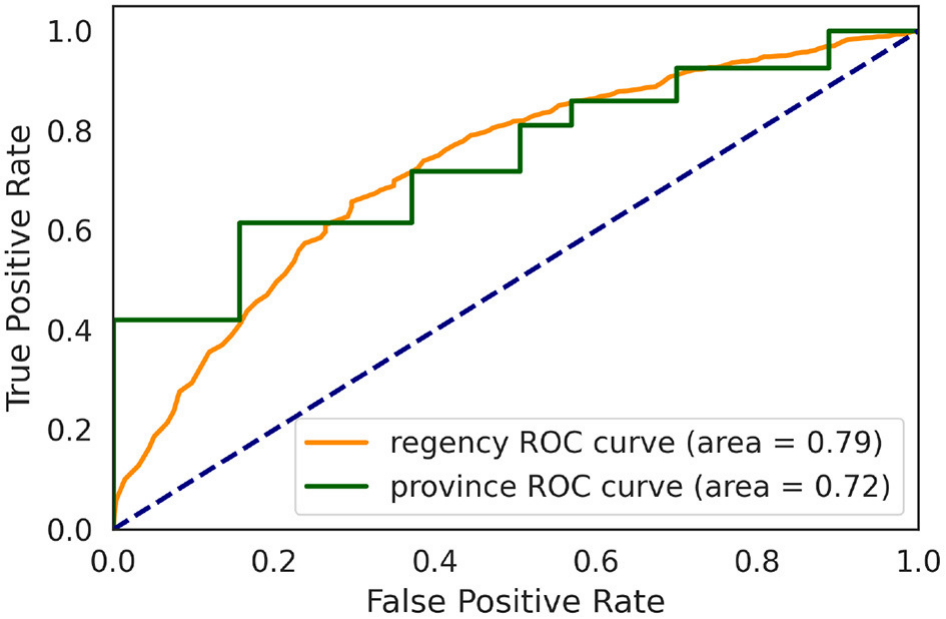
ROC curve of the prediction results using SUSENAS index as ground truth. Each point is obtained by progressively increase the target population by 1% at a time. The dashed blue line shows the performance of the baseline classifier. The green line shows the prediction result of the RWI map on data aggregated at the province level while the orange line shows the predictions of the RWI map on data aggregated at the regency level.The population density of the Indonesian provinces creates a clear ladder shape. Clearly, targeting at a more refined level leads to a better performance.

A common criticism of the geographical targeting focuses on the heterogeneity of socioeconomic conditions within a geographic unit; hence, the level of spatial aggregation is a determining factor. Following a similar methodology to a preprint of one of the RWI map's authors,^8^ we evaluate the predictive power of the RWI map by performing the targeting at a regency level but evaluating the precision at a household level against the SUSENAS index. In this way, we compare the two indices at their lowest spatial aggregation precision. Figure 3 depicts the different performances at province and regency levels, measured as the area under the receiving operating characteristic (AUROC), improving from 0.72 at the province level to 0.79 at the regency level.

It has to be remembered that we are comparing only the match between indices, not which one is better at predicting the monetary poorest. Secondly, while the difference is statistically significant, it may not be substantively significant (McCloskey and Ziliak, 1996). In other words, the precision may be higher but the magnitude of the difference may be small. Thirdly, in such a case, the higher prediction should be evaluated against the social costs of the finer distinction among households within a community.

### 3.3. Targeting for cash transfer

**Figure 4:**
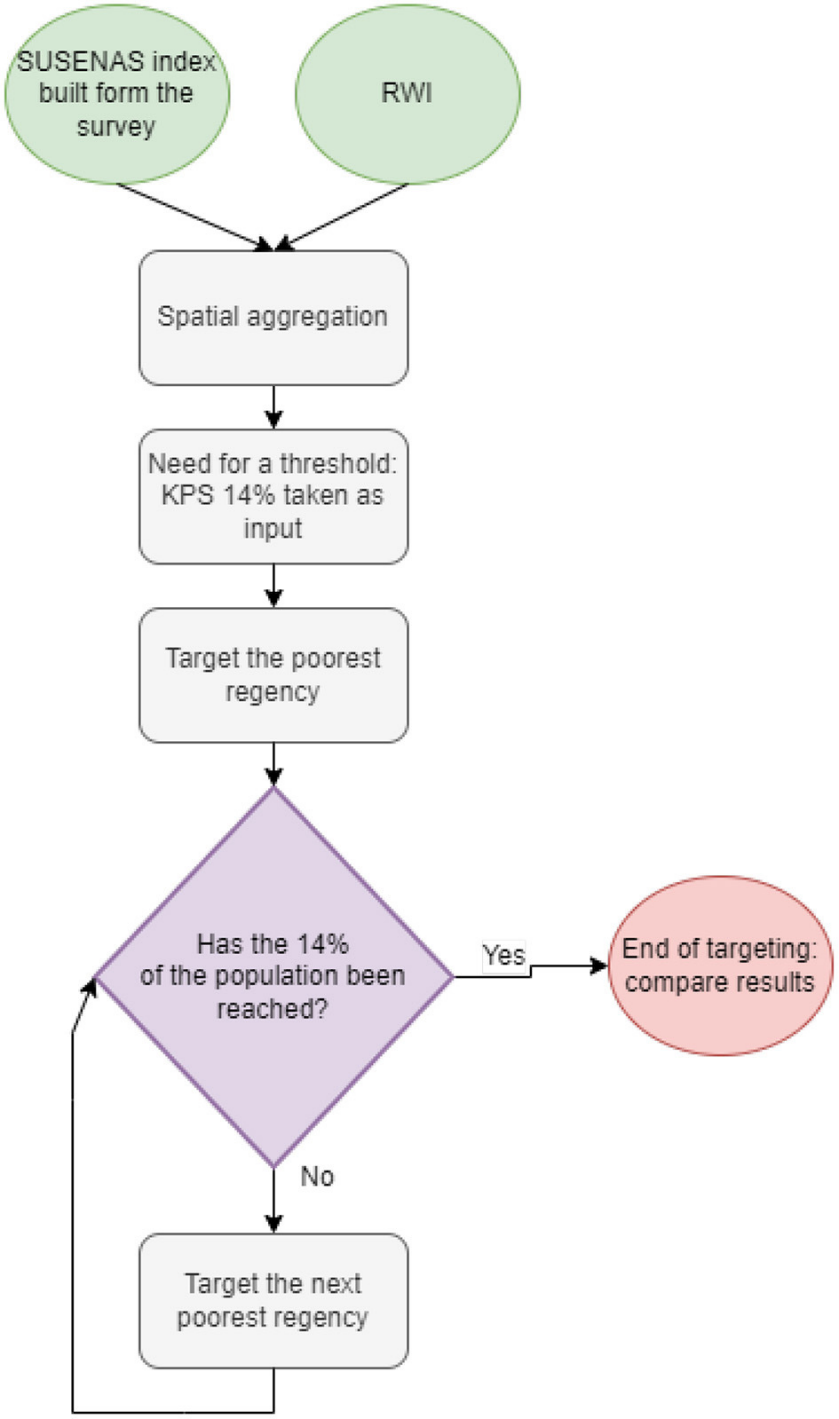
We show the pipeline to target the 14% poorest, starting for the wealth indices. Once the population threshold is fixed, regencies are iteratively targeted, starting from the poorest one, until the desired threshold is reached.

We explore the possibility of using the RWI map as a targeting tool using as a guideline the specifics of an assistive initiative of the Indonesian government, the KPS (Kartu Perlindungan Sosial) program. For the sake of clarity, in this exercise, we use the KPS guidelines only to choose a proper poverty threshold and consider the 77(14%) poorest regencies in the country. This is due to a limitation in the RWI data resolution, which is only available at the regencies level, and we cannot have a proper estimate of the poverty threshold at a household level. The targeting workflow is shown in Figure 4.

#### 3.3.1. Population bias

**Figure 5:**
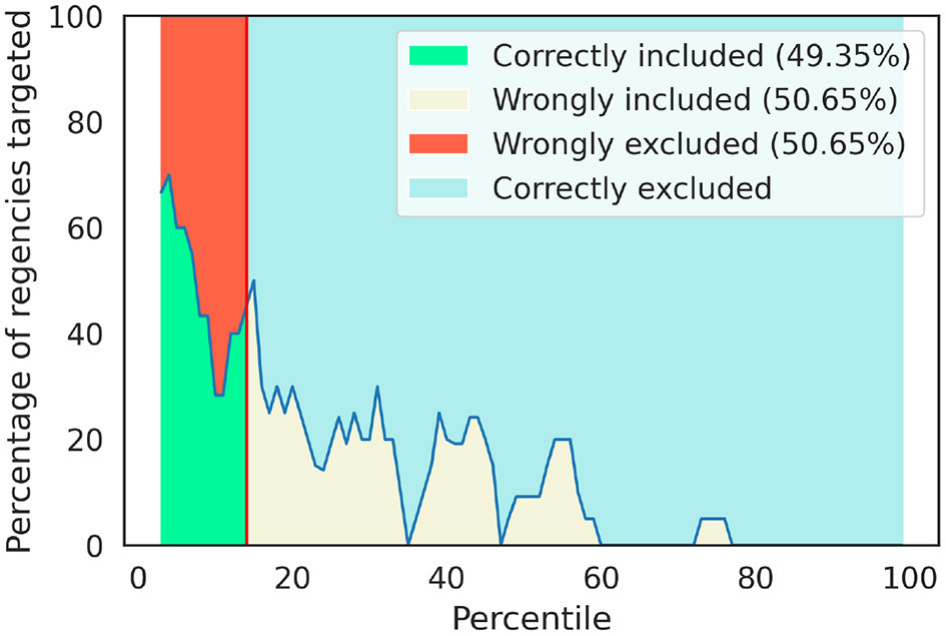
Distribution by percentile of the 14% poorest regencies according to the RWI map, as labeled employing SUSENAS index as ground truth. The vertical red line signs the 14^*th*^ percentile. The correctly included regencies are highlighted in green, while the exclusion error is in red and the inclusion error is in beige. We notice that these two errors are equal since there is a wrongly included one for every wrongly excluded regency. A perfect targeting strategy would be a perfectly horizontal line at the 100% mark before the vertical red line and a straight line at the 0% mark after. It must be noted that the percentage of wrongly excluded regencies must be equal to the number of wrongly included ones, which is equal to 100% minus the percentage of correctly included regencies.

**Figure 6:**
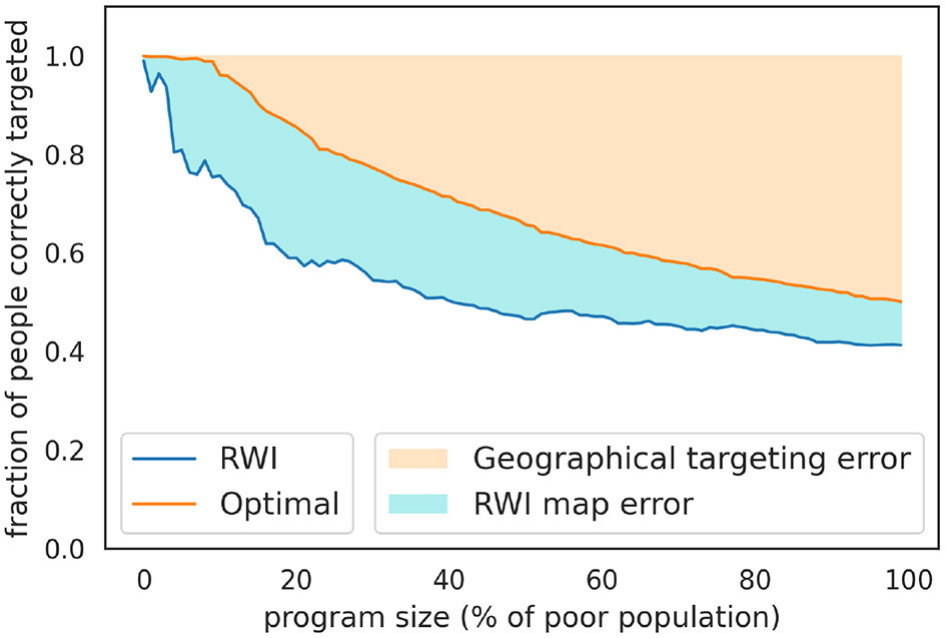
Percentage of people correctly targeted by the RWI map and the SUSENAS index for the KPS program, benchmarking against the SUSENAS index. The targeting is done by aggregating the asset-based indices at the regency level. Since the ground truth results are the best attainable, they are labeled as “Optimal” within the graphs. It must be noted that even these are not perfect since geographical targeting has an inherent error due to the heterogeneity in wealth inside administrative areas. This intrinsic error is highlighted in light orange, while the error we would make by choosing the RWI map is colored in light blue. When targeting more than ~30% of the poor population, we notice how the second source of error is less significant than the first one.

We confront the tile-by-tile targeting by employing the RWI map to predict where the poorest 14% of the population resides. Interestingly, the agreement between the SUSENAS index and the RWI map is on just 38(49.35%) of the regencies (Table 1). It is also important to note that some of the areas predicted by the RWI map as some of the poorest are labeled by the ground truth as among the wealthiest half of the country. The exclusion error reaches the 39 of the regencies comprising about half of the territory 50.65% (inclusion error of 50.65%), respectively. In Figure 5, we show the percentile distribution of the regencies that, according to the predictions of the RWI map, are eligible for the KPS benefits. Out of the total population living in the eligible regencies according to the SUSENAS index, the 55.66% would not be targeted by the RWI map (Figure 6).

**Table 1:** KPS targeting descriptive results for both the regency-based (spatial) and the population-based approach respectively.

	**Spatial targeting**	**Population targeting**
Precision	49.35%	64.91%
Number of FN	39	74
Population in FN	7.5 mil	17.7 mil
Exclusion (FN)	50.65%	40%
Exclusion (pop)	55.66%	32.82%

The results were obtained by employing RWI map with SUSENAS index as ground truth.

To address these inequalities in representation, we implement a population-related definition of the threshold to refine the targeting strategy. We iteratively target the poorest regencies until reaching ~36 million people, which represents approximately the 14% of the Indonesian population. We employ the SUSENAS index as the ground truth and directly compare it to the RWI map predictions. We find that the percentage of wrongly excluded regencies drops to (40%), while we recover a 32.82% of the poor population RWI wrongly excluded from the program.

The targeting according to this population-defined threshold can also be carried by the SUSENAS index at the household level, giving us a precise percentage of the eligible people to be targeted within each regency instead of a generic estimate of the population living in the area to be targeted.

#### 3.3.2. Geographical bias

**Figure 7:**
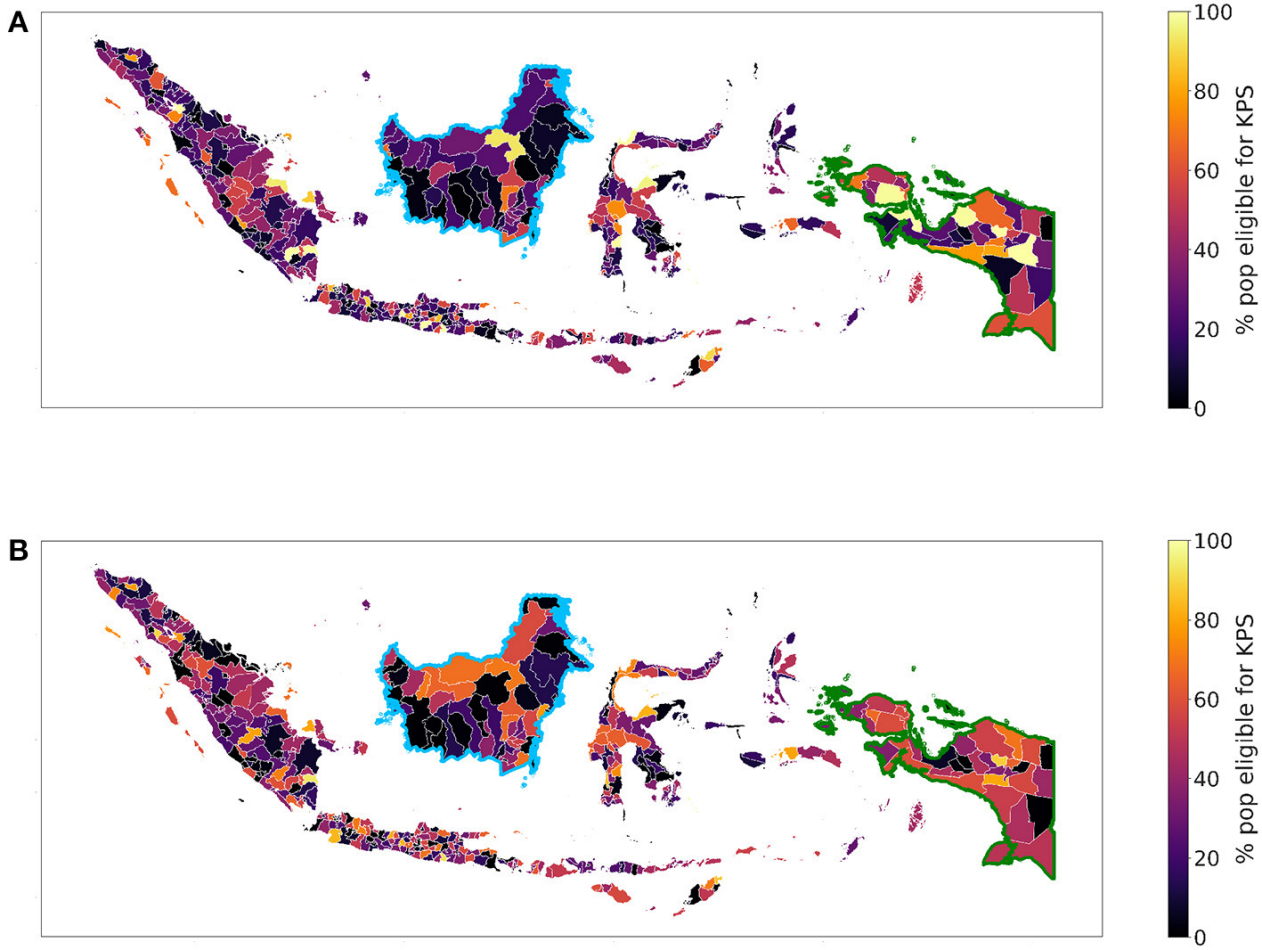
The maps show the percentage of people eligible for the KPS program (i.e., belonging to the 14% poorest) according to the **(A)** SUSENAS-engineered wealth index or **(B)** RWI map estimates, aggregated at regency level. A higher percentage corresponds to a poorer regency. In both maps, the Kalimantan region is highlighted by the blue border and the Papua region by the green border.

**Figure 8:**
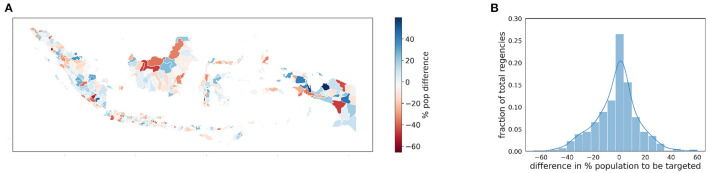
The map **(A)** shows the distribution spatially, regencies colored in blue are under-targeted by the RWI map, while red regencies are over-targeted. The histogram **(B)** shows the distribution of the difference percentage of people eligible for the KPS program (i.e., belonging to the 14% poorest) between the estimates of the SUSENAS index and the RWI map respectively.

Because of the specifics of the KPS program, we consider the Indonesian population living in monetary poverty. For the SUSENAS index and the RWI map, we estimated the percentage of people in each regency's lowest range of the asset wealth index distribution. Lacking geographically precise estimates, we employ the household information provided by the SUSENAS index as a term of comparison. The obtained maps for SUSENAS and RWI are depicted in Figures 7A, B, respectively. We notice utter differences between the two indices when computing the percentage differences of the population to be targeted (Figure 8). In particular, we observe this divergence for the Kalimantan (highlighted in the figure with a blue border) and Papua (green border) regions, with the latter being the country's poorest region according to the SUSENAS ground truth. These results are similar to the ones mentioned in Section 3.2. Given the volatility of the SUSENAS wealth index and the different indicators and methods in the RWI, the such discrepancy should not be surprising.

## 4. Limitations

Our analysis has several limiting factors aside from the ones already mentioned. The nature of the index (asset-, expenditure- or income-based) focuses on specific facets of poverty, a truly multidimensional quantity. The literature on monetary poverty targeting in low and medium-income countries are usually estimates of expenditure (Simler and Nhate, 2005), or assets belonging to the households (Kaiser et al., 2017). However, these two definitions are not at all interchangeable (Howe et al., 2009). The RWI Filmer-Pritchett pipeline involves a series of arbitrary decisions introducing errors. One of the main shortcomings is the construction of the wealth index (Gordon and Nandy, 2012) for the comparison. Different implementation choices in index creation lead to very different wealth indices. As the last point, here, we focus on geographical targeting since the RWI map is built for this scope; however, different methodologies such as self (Alatas et al., 2016), or community-based (Alderman, 2002) targeting are also commonly used.

## 5. Concluding remarks

Population surveys are of fundamental importance for understanding society and devising better and more efficient ways to combat social inequalities. Without their detailed and representative (for the country and subnationally) results, the magnitude and characteristics of social problems would not be known. However, it is well-known that implementing programs, unlike social analysis, requires different data (e.g., administrative data). The enormous achievements of machine learning are increasingly influencing other disciplines, including social sciences and demography, and it is reasonable to explore if they could be used for program design and implementation. Novel tools based on machine learning come to fill in the gaps of conventional sources; however, they entail a limited accounting of intrinsic biases in the training data, which should be instead properly assessed with caution to avoid introducing or augmenting social inequalities (Beiró and Kalimeri, 2022).

This study contributes to addressing the concerns of practitioners and policymakers regarding the trustworthiness of a machine learning-based index for social assistance. Focusing on Indonesia, we systematically compare the RWI index (Chi et al., 2022), a machine learning-based index inferring on social media and satellite imagery data, and the SUSENAS index, a relative poverty estimation index emerging from the respective national-wide survey. We do so by assessing the sociodemographic impact of a hypothetical scenario based on an assistance program designed by the Indonesian government, the KPS. The aim is to highlight the strengths and limitations of employing an ML-based tool such as the RWI to guide program implementation.

Initially, our analysis pointed out several shortcomings in constructing survey and machine-learning-based indices. The theoretical limitations of the Filmer-Pritchett wealth index (Gordon and Nandy, 2012), together with the sparse availability of fine-grained survey data, lead to questionable policy making decisions. Here we examined both time and spatial aggregation effects of the survey-based index, showing that the socioeconomic profile of a country changes over time; hence, ML-based indices should be retrained to reflect an up-to-date view of the society. The spatial aggregation was also shown to amplify existing issues in directly benchmarking ML-based estimates to the official survey. In our case, the RWI index was trained on very high geographical precision data, which are not available in the more recent waves of the official surveys; hence, we aggregated at a higher administrative level to perform a direct comparison. We highlight under and over-representation issues emerging from the aggregation of socioeconomically heterogeneous regencies for a direct index comparison.

By applying those indices to inform a hypothetical targeting for the KPS program, we demonstrated how the biases emerging from the temporal deterioration and the spatial aggregation have critical social implications. We show that the exclusion error of the RWI index impacts about half of the territory 50.65% (inclusion error of 50.65%), respectively. In contrast, out of the total population living in the eligible regencies according to the SUSENAS index, the 55.66% would not have been targeted. Despite the limitations of the approach, our results show that the RWI index is sensitive to socioeconomic information's time and space variability. This is particularly interesting because ML-based indices, such as the RWI, are appealing for policy-making due to the immediate and cost-effective estimates they can provide. With this analysis, we want to highlight that a potential direct application of the RWI index to a real-world scenario would be sensitive to the socioeconomic particularities of the country, leading to significantly different estimates from the ones obtained by a traditional survey approach. The goal of this analysis is again to stress how any specific policy-making decision has to be implemented, taking into account the complexity of each country's socioeconomic context and relying on a diverse set of data, methodologies and approaches.

## Data availability statement

The data analyzed in this study is subject to the following licenses/restrictions: Data cannot be shared without the permission of the data provider. Requests to access these datasets should be directed to daniela.paolotti@isi.it.

## Author contributions

DS, KK, MG-H, ED, DP, and RS conceived and planned the experiments and contributed to the interpretation of the results. DS carried out the experiments. DS, KK, DP, and RS took the lead in writing the manuscript. All authors provided critical feedback and helped shape the research, analysis, and manuscript. All authors contributed to the article and approved the submitted version.

## Footnotes

^1^Source Asian Development Bank.

^2^These long-term comparisons are invalidated by changes in production and consumption patterns that make the original poverty line irrelevant.

^3^World Bank (2018).

^4^The map is publicly available on the Humanitarian Data Exchange (HDX) website.

^5^Individual elements considered: “electricity in household, telephone, automobile, motorcycle, refrigerator, TV, water supply, cooking fuel, trash disposal, toilet, floor material, wall material, roof material and rooms in house.” The asset “Radio” is missing from the SUSENAS survey, so we skipped that element.

^6^The detailed process is described in Facebook's tutorial https://dataforgood.facebook.com/dfg/docs/tutorial-calculating-population-weighted-relative-wealth-index.

^7^We consider the 14% poorest percentiles in line with the Kartu Perlingudan Social (KPS) program.

^8^ www.jblumenstock.com/files/papers/SmytheBlumenstockPreprint.pdf
